# Risk factors associated with the family care of people with serious mental illness

**DOI:** 10.4317/medoral.23133

**Published:** 2019-06-25

**Authors:** Ángela Carbonell, José-Javier Navarro-Pérez, Maria-Vicenta Mestre

**Affiliations:** 1 Department of Social Work and Social Services, University of Valencia (Spain); 2 Inter-University Research of Local Development (IidL), Department of Social Work and Social Services, University of Valencia (Spain); 3 Department of Basic Psychology, University of Valencia (Spain)

## Abstract

**Background:**

The aim of the present study is to analyse the variables associated with the family care of people diagnosed with serious mental illness.

**Material and Methods:**

A cross-sectional study was carried out involving caregivers of people with serious mental illness (SMI) who were known to the mental health services in Valencia (España) and associations for those with SMI. The sample comprised 417 caregivers who completed a sociodemographic questionnaire and the Zarit Burden Interview. Bivariate analyses (t-test, analysis of variance and Pearson correlation) were performed, as was a multiple linear regression model. Values of *p*< .05 were considered significant. The study was carried out in accordance with the recommendations of the ethics committees of the participating institutions.

**Results:**

The statistical analyses showed significant associations between the sociodemographic and clinical variables of the caregivers and patients and the burden felt by caregivers of people with SMI. The importance of both formal and informal social support stands out as a protective factor against the consequences of the illness’s impact on the main caregiver.

**Conclusions:**

The role of spaces of mutual support is crucial. The results suggest that family psychoeducational programmes should be created, applied and evaluated in all mental healthcare services so as to reinforce training in mental health matters and provide support and assessment to caregivers in order to ease their burden.

** Key words:**Care, social support, mental health, associationism.

## Introduction

Mental healthcare has undergone far-reaching epistemological and practical changes, representing a long and complex historical and cultural process. Indeed, the World Health Organization has for many years been questioning the role of psychiatric institutions and healthcare processes dealing with mental health. Until the mid-twentieth century the only treatment provided by psychiatry was hospitalization for indefinite periods of time. The appearance of new community healthcare models for people with mental illness, based on criteria of quality of life and decent care, and the incipient development of psychopharmacology marked the beginning of a move towards psychiatric deinstitutionalization (1) involving the closure of psychiatric hospitals and the release of people with serious mental illness (SMI) into the community (2).

Despite the fact that the mental healthcare system in Spain has fought to provide the necessary support services, the family continues to be the main source of care for people with SMI. Although family care can have a positive impact on the rehabilitation and recovery of these people, the scientific literature is rich in studies that show that their caregivers can experience strong feelings of burden and objective and subjective distress (3) as a result of their many responsibilities and the exhaustion these induce. Apart from the mental and emotional impact, the care burden involves anxiety-provoking aspects such as economic cost, shame, stigma and feelings of guilt and self-pity.

Taking into account the negative repercussions of psychiatric family care, studies have analysed how the care burden can be influenced by the sociodemographic and clinical characteristics of the patients and caregivers and how these affect the caregivers’ quality of life (4). The feminization of care makes it difficult to provide significant evidence of the burden according to gender (5). Nevertheless, it is the mother who usually performs the task as a moral obligation in a patriarchal society, which makes it difficult to reconcile work and care. A study carried out in Spain showed that the parents of sick people feel greater levels of burden and worry more about their future than other family members (6). Studies have associated the lengthy nature of chronic illnesses and the ageing of caregivers with an enormous family burden, revealing higher levels of burden in caregivers whose family members have been sick for a long time or whose illness began at an early age, and with less autonomy and more active symptoms that require a greater number of hospitalizations (7,8). Other demographic characteristics of the caregivers, such as economic income and level of education, have been shown to correlate with their psychological and physical burden. Social support, however, is considered to be a protective factor (9) since it makes it possible to understand the compensation and adjustment mechanisms used to deal with the difficulties of care and to reduce the harmful effects that emerge as a result of providing continuous care for someone.

The aims of this study are (1) to identify the characteristics of the caregivers of people with SMI, (2) to identify the level of burden, and (3) to analyse the variables associated with family care in mental health.

## Material and Methods

A descriptive cross-sectional study of family caregivers of people with SMI was carried out.

-Participants

The study population consists of family caregivers. The inclusion criteria were (1) resident in Valencia (España), (2) caregiver of a person diagnosed with an SMI according to the DSM-5 and responsible for the associated tasks, (3) age ≥ 18 years, (4) no psychiatric history, (5) family member or relative, (6) care ≥ 6 months, (7) absence of remuneration for the care provided, and (8) voluntary participation in the study. Those who did not complete the questionnaires correctly and those who declined to participate were excluded. 417 family caregivers of people with SMI were included as participants in the study.

-Instruments

The main instrument used was the Zarit Burden Interview (ZBI) (10) as adapted into Spanish by Martin *et al.* (11), which measures the extent of the caregiver’s burden. Although this has mainly been used with the caregivers of people with dementia, the extensive literature (12,13) endorses its use also with caregivers of people with mental illness. It consists of 22 items recorded using a Likert scale from 0 (never) to 4 (nearly always). The results add up to a total score of between 0 and 88 points. Higher scores indicate greater caregiver distress. For the present study the scale obtained a Cronbach’s alpha coefficient of .91.

Included were the sociodemographic characteristics of the caregivers (sex, age, marital status, relationship to the sick person, employment situation, presence of health problems, active associationism, psychoeducational interventions and geographical setting) and of the people cared for (age, sex, diagnosis, number of years since SMI was diagnosed, degree of disability and care received).

-Procedure 

The participants were recruited via mental healthcare facilities and associations of patients and family members of people with SMI in Valencia (España). An appeal was made for volunteers and information about the study was provided. Once the participants had been selected, individualized interviews were arranged, in which the Spanish version of the ZBI questionnaire was administered and sociodemographic and clinical details collected. Participants received no monetary compensation for taking part in the study. Data were collected between June and December 2018.

The study was carried out in accordance with human rights protection protocols and satisfied the ethical requirements for research approved by the institutional review board before the participants were recruited. The family caregivers were sent a letter that explained the details of the study and informed them that they had the right to interrupt or leave the study at any time and for any reason, in accordance with the Declaration of Helsinki. All participants gave their informed consent in writing before data were collected.

-Data analysis

The IBM SPSS Statistics 25 package was used for the data analysis. The level of statistical significance was established as *p* < .05. Descriptive statistics (percentages, means and standard deviations) were used to describe the sociodemographic characteristics of the caregivers and the people being cared for. The relation between sociodemographic characteristics and burden was identified. Inferential analysis involving t-tests and ANOVAs was used to identify statistically significant differences between the ZBI and the nominal variables. Correlations between continuous variables were analysed using the Pearson correlation coefficient. Finally, those variables that showed a statistically significant association were included in a stepwise multiple linear regression analysis to determine the predictive factors for caregiver burden.

## Results

-The participants’ sociodemographic characteristics

The study sample comprised 417 family caregivers of people with SMI, 72.2% of whom were women. Ages ranged between 18 and 89 years, with an average of 60.82 (SD = 13.46). Generally speaking, the participants in the study were the parents of those looked after (78.7%) and were married (52.8%) or divorced (14.9%). Most were not in paid employment (59.7%) and 49.2% had previously been caregivers of another sick person. The participants’ sociodemographic characteristics are listed in  Table 1.

**Table 1:**
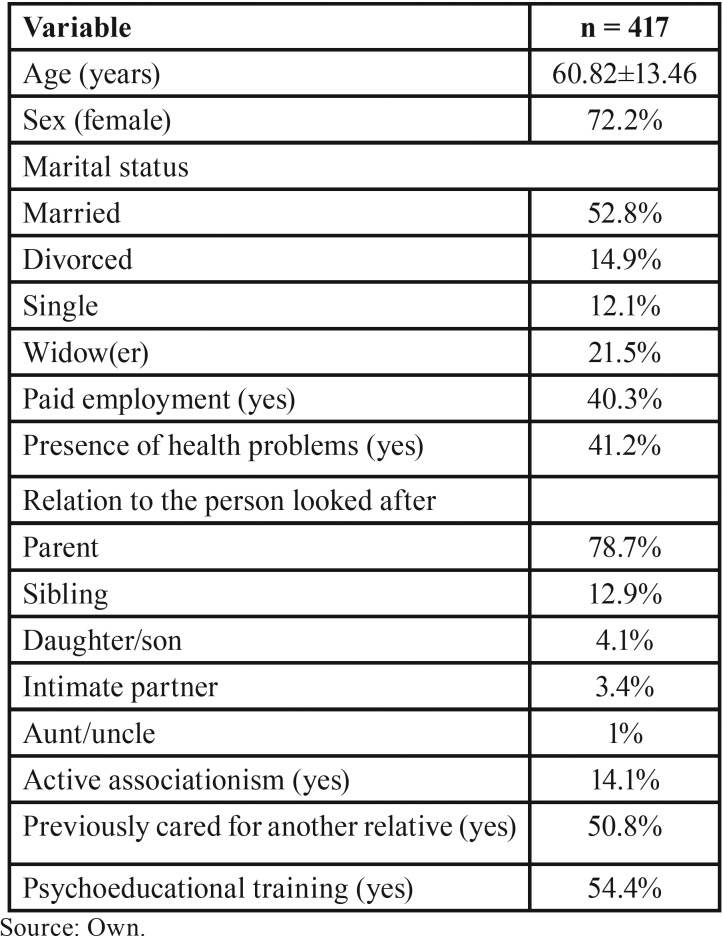
Sociodemographic characteristics of the study participants.

The general profile of the person receiving care was male (69.8%) with an average age of 38.78 years (SD = 12.88; range = 16-80 years). All those being looked after had been diagnosed with an SMI – according to DSM-5 criteria – by psychiatric staff belonging to the public healthcare services in Valencia (España). The most common diagnoses were schizophrenia (62.1%), personality disorder (14.9%) and bipolar disorder (12.2%), and patients had had the illness for an average of 17.22 years (SD = 13.08). In addition, 49.4% had a recognized disability that affected their personal autonomy to a high degree.

-Caregiver burden and associated variables

The overall average score for burden was 45.79 (SD = 17.09, range 10-80), with 14% of caregivers registering no burden at all. The rest showed light (24.9%) and intense (61.1%) levels.

Comparison of means tests indicated there were variables with values of statistical significance *p* > .05, which included marital status, having a chronic illness, having previously cared for someone else, and family relationship. The analysis established differences depending on the participants’ sex and care burden (t = 1.932; *p* < .05), with men obtaining an average score of 48.53 (SD = 18.06) and women 44.76 (SD = 16.64). Caregivers in paid employment felt significantly higher levels of burden (M = 49.96; SD = 17.44) than those who were not active (t = -4.133; *p* < .05). Lower levels of burden were associated with caregivers who had taken part in psychoeducational interventions (t = 12.978; *p* < .05) and/or were members of an association (t = 13.724; *p* < .05). Geographical setting was also significantly related with the caregiver’s burden (t = -5.962; *p* < .05), with caregivers living in metropolitan and rural areas obtaining an average score of 50.04 (SD = 16.33) compared to the 40.32 (SD = 16.55) of those living in towns and villages.

As far as the variables for the person looked after are concerned, the t-tests indicated that there were differences in the level of burden depending on their sex (t = -2.096; *p* < .05), with caregivers who looked after women showing higher levels (M = 48.37; SD = 16.14) than those who looked after men (M = 44.66; SD = 14.41). Similarly, the caregivers of people who received continuous care in public mental healthcare facilities had an average burden score of 33.29 (SD = 14.143), while those who looked after people who did not had an average of 51.97 (SD = 14.93) (t = -12.411; *p* < .05). The descriptive inferential analysis found that the mental illness diagnosis of the person looked after also had a significant effect on the caregiver burden, F (6,413) = 3.974, p < .05, η² = .056), with very high levels being found in caregivers of people with personality disorder (M = 54.31; SD = 12.49) compared to other diagnoses such as schizophrenia (M = 43.27; SD = 17.14).

Pearson correlation analysis ( Table 2) found negative associations between the care burden and age (r = -.147; *p* < .05), with levels of burden diminishing as the caregiver’s age increases. No significant relations were found for the disability and age of the person cared for. However, the years of duration of the illness correlated positively with burden (r = .175; *p* < .05).

**Table 2:**
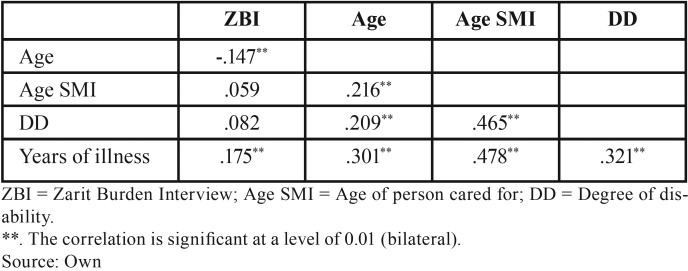
Pearson correlations for the dimensions of the variables analysed.

To determine which variables contributed with significant unique predictive variance, the significant predictors were included in a multiple linear regression. As can be seen in  Table 3, six regression models were constructed, revealing that the factors significantly associated with the highest burden scores were (1) participation in psychoeducational interventions, (2) associationism, and (3) continuous healthcare. The regression was highly significant (adjusted R2 = 0.41; F = 3, 97.214; *p* < .05).

**Table 3:**
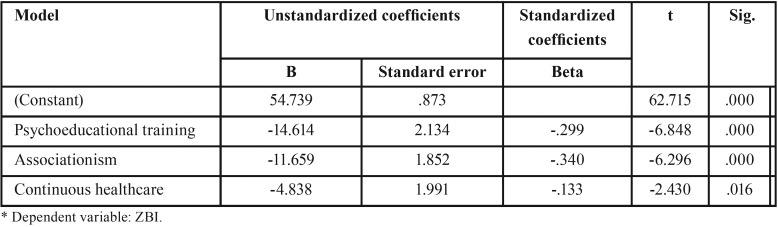
Linear regression of the burden of the family caregiver of people with SMI.

## Discussion

The family is the main support system and takes on the responsibility of caring for the patient in the community. The present investigation provides new evidence of the magnitude of the care burden, identifies factors associated with it and analyses the sociodemographic profile of 417 family caregivers of patients with SMI treated in different healthcare services in Valencia (España)

As far as the study’s first aim is concerned, the sociodemographic characteristics of the sample are similar to those in other studies on caregivers of patients with SMI (14,15), in which the predominant profile is that of a woman just entering old age, married, without paid employment and with no chronic pathologies. Considering this, it is clear that there is inequality in the provision of care since it is mainly carried out by women, and this makes these women a collective that is vulnerable to the consequences resulting from the work they do. 72.2% of care is in the hands of women. Although the results of this study indicate that it is more usual for them to take on the task of caring (M = 44.76), the men obtained a higher average score (M = 48.53) for burden. Meanwhile the profile of the person with SMI matches that in Kate *et al.* (16): male, adult, diagnosed with schizophrenia, living with parents, and with the mother as the mainstay of care and attention.

The analysis of caregiver burden produced an average score of 45.79, and three-quarters of the population surveyed registered intense levels of burden on the ZBI, revealing that the caregivers of people with SMI felt a significant burden similar to that found in other studies in the literature (17,15), which warn that this population could be at risk of being overwhelmed.

In this study the caregiver burden is associated with sex and age, with the highest levels being found among male and younger caregivers, unlike in other recent investigations (18,19) that indicate that looking after women and older patients are sources of stress and factors predictive of burden. Following Blanco *et al.* (20), this could be because inexperience and the opportunity costs of care may cause a feeling of burden in younger male caregivers. Another factor associated with caregiver burden was the problem of reconciling working life with care (21). Both the existing literature (22) and the results of this investigation describe characteristics typical of SMI (diagnosis, prognosis, years of illness, etc.) as being factors that induce feelings of burden in the caregiver. Hence the findings relate disorders with more complex symptoms (such as personality disorder) and the years of duration of the illness with greater levels of distress in the caregiver.

Despite the fact that the literature has focused mainly on the pathogenic factors of care, the results of the regression analysis showed that the variables connected with formal and informal social support were the most important mediator variables for caregiver burden (4,23). Access to continuous specialist healthcare, associationism and psychoeducational interventions for family members determined the burden levels of the caregivers, working as protective factors against the stressful impact of providing care. As argued in earlier studies (16,23,24), caregivers who perceive that they obtain sufficient support from institutions, family members and friends have a better quality of life and feel less distress in connection with the work they do.

-Limitations

The present study is subject to two limitations. The cross-sectional nature of the investigation made it possible to establish a predictive model for burden. However, longitudinal studies are more suitable for verifying the predictive factors of caregiver burden. Another important limitation was the non-existence of specific resources aimed at the caregivers and family members of people with SMI. This also meant that the sample was obtained in two blocks: from family associations of people with SMI and the public mental healthcare services whose family members agreed to participate in the study.

Despite the above limitations, this study has revealed a significant relationship between the sex, age and employment situation of the caregivers, the age and diagnosis of the patient, the years of duration of the illness and the caregiver burden of those looking after people with SMI. Social support stood out as a protective factor for care in a wide, representative sample of caregivers, allowing interventions to contribute to lessening the burden felt by the caregivers of people with SMI.

Today the support the public system gives is not enough. Although the government does provide resources for people with SMI and their family members, these resources are still limited and poorly funded. Families stand in for the lack of public resources earmarked for people with SMI. Thus patients remain with their families and this makeshift arrangement becomes an end in itself, even though the actual root of the problem is not tackled. Instead, problems are dealt with as they appear. The family is not given even the minimum resources to provide suitable care in each case. The consequences of informal care for the people that provide it continue to be a common challenge, and the response needs to involve the creation of real integrated healthcare policies aimed at both people with SMI and their caregivers. The state should promote public policies providing real support for family members who perform these tasks for other family members. This support should include rehabilitation services, psychosocial care, education, and training in carrying out both care and self-care tasks, and be provided not only through the mental healthcare services but also via other psychosocial healthcare facilities.
